# Opportunistic Risk Screening of Depression by Community Pharmacists: Noble Intervention to Mend the Mind during COVID-19

**DOI:** 10.21315/mjms2022.29.4.15

**Published:** 2022-08-29

**Authors:** Ramasamy Chidambaram

**Affiliations:** Department of Prosthodontics, Faculty of Dentistry, SEGi University, Selangor, Malaysia

**Keywords:** community pharmacists, community pharmacies, depressive symptoms, family physicians, pandemics

## Abstract

Depression is the most common condition affecting the mental health of individuals. During the whole lifetime, at least one in four individuals go through an episode of this disorder. Recently, the Malaysian number of cases has increased to around 500,000 with more adding due to COVID-19 pandemic. The first professionals to detect depression by analysing the rather emotional, presenting symptoms of the patient are the family physicians. In such circumstances, how is it possible for a community pharmacist to identify undiagnosed depression? Depression is real, sadly, the silent nature of the disorder results in an increase in its unreported cases. On the bright side we believe the new screening guidelines and intervention of community pharmacists might be one noble way to aid in the opportunistic risk screening of depression and heal the hidden emotions during COVID-19.

## Introduction

Depression is the most common condition affecting the mental health of individuals. During the whole lifetime, at least one in four individuals experiences this disorder (1). The first professionals to detect depression by analysing the rather emotional, presenting symptoms of the patient are the family physicians (2). They are in fact the first specialists to identify and treat a state of illness. Given that, the critical question is that how is it possible for a community pharmacist to identify undiagnosed depression? Probably readers could raise the question that targeting the prevention of the modifiable risk factors might reduce its prevalence. Depression is real, sadly, the silent nature of the disorder results in an increase in its unreported cases. According to the National Health and Morbidity Survey, 2.3% of the Malaysian adult population experienced depression (3). Recently, the number of cases has increased to around 500,000 with more adding due to COVID-19 pandemic (4). The unexpected trouble, COVID-19, with zoonotic background has affected the healthcare systems worldwide. In current statistics while writing this communication, 174.9 million cases were found confirmed subjects and sadly 3.5 million had succumbed to death (5). In the same context, 673,206 cases including 4,142 deaths have been reported in Malaysia (6). On the bright side, the new screening guidelines and intervention of community pharmacists is one noble way to recognise depression and heal the hidden emotions during COVID-19.

### The Community Pharmacist’s Profession

The third-largest devoted healthcare professionals in the world are the community pharmacists. Compared to patient visiting a physician in a year, the number of times patients pay visit to community pharmacist is much more (nearly 12 times) (7). The given evidence further reinforces an opportunity for community pharmacists to recognise depressed patients. Most diseases are diagnosed with a test. There are no tests to detect depression as such. Monitoring of patients consuming medications that may predispose them to symptoms of depression, as a side effect, is one way by which community pharmacists can help in the screening of depression. Patients consuming such medications are the ones who are generally affected by more than one chronic disease such as diabetes, cancer and heart disease, which, in turn, may cause depression, thereby decreasing and affecting their quality of life (8–9).

Like other countries, COVID-19 had made an impact on Malaysia (10). A collaborative study of seven Asian countries conducted on 4,479 candidates suggests that along with Pakistan and Thailand, Malaysia is one among them to secure high Depression Anxiety Stress Scale scores (11). In the same context, a cross-sectional study was conducted involving several Malaysian cities, in which around 326 candidates’ anxiety were dominant (41.7%) and nearly one-third showed depressive symptoms (12). The remaining participants had mood disorders. The rural areas showed scary pattern of depression accounting to increased risk of 180% (13). The results show a clear picture of higher anxiety and depression rates post-pandemic owing to economic and social crisis. However, professional screening is the gold standard. Screening programmes, particularly opportunistic screening services, can suitably be carried out in community pharmacy, facilitating early diagnosis of depression. It is the fact that the person next to you might have experienced depression in his/her entire lifetime. A community pharmacist has ample opportunities to communicate with the patients and build a healthy rapport with them as patients generally need to drop off, pick up and wait for their prescription to be filled, thus spending a long time with the community pharmacists. Though for them it’s easy to have causal communication with patients, yet such a trivial exchange of ideas proves to be futile in recognising the hidden concerns linked to depression.

Depression, from a patient’s perspective, is not discussed substantially. Hidden beliefs and myths that underlie the stigma create barrier to diagnosis. Understanding the view about depression from patient’s own words will motivate to accept the offered service (screening) and mutually avert potential coercion. In good will, seeking written consent from the candidate would enable to develop mutual trust. Training programmes must be conducted to impart the knowledge in community pharmacists and advise the patients on depression and antidepressant medications. Accordingly, a community pharmacist must have the skill set needed to detect the poor state of mind and a pre-prepared questionnaire.

### The Community Pharmacists Tools

Indices like Patient Health Questionnaire (PHQ) and Well-being Index can conveniently be used for screening patients with depression (14). Nevertheless, it must be pointed out that the numerous depression screening tools, though readily available, differ widely in terms of their psychometric properties. Interestingly, in a recent systematic review, it was found that PHQ-9 is the most extensively surveyed depression screening tool, among the 55 tools, in view of its feasibility, reliability, validity and practicality (15). In the light of this exhaustive review, reliability and validity of PHQ-9 can be accepted. The highlight being, it offers the highest accuracy and mutually increases the diagnostic possibilities for a community pharmacist. Meanwhile, the new recommendations of the United States Preventive Services Task Force (USPSTF) suggest that all adults, including pregnant and postpartum women, should undergo screening for depression. According to their guidelines, PHQ questions 1, 2 and 9 (Table 1) are those that determine patients who require assistance with depression and therefore it is commonly referred to as ‘PHQ 2+1’ (16–17). As per our understanding, the update to screening recommendations is a potential opportunity for community pharmacists to step up and recognise high-risk individuals who require further evaluation and treatment for depression. However, this is not the only role of community pharmacists; in fact, these processes are considered successful only when community pharmacists take the necessary actions and refer the suspected individuals to family physicians. Community pharmacist may take permission from the suspected candidate to seek mental health professionals’ (psychiatrist/clinical psychologist) advice where pharmacological intervention serves as effective modality.

### The Community Pharmacists Growth

The available literature gives us mixed results in which a cross-sectional study using depression vignette among 200 registered community pharmacists found that more than half of the respondents identified the disorder (18). Meanwhile, the researchers with positive finding recommend that budding pharmacists require special training to liaise with experienced people. A similar study was conducted on 96 community pharmacists, which resulted that only around one-third of them had adequate mental health literacy (19). From the Ministry of Health Malaysia’s corner, ‘Benchmarking Guideline for Community Pharmacists’—updated periodically—serves as a set of rules and regulations that sustain the standards of community pharmacy practice (20). The elaborative report does not fail to quote that community pharmacists are entitled to offer advice on health-related matters wherever required. This does strengthen the undertaken communication as well as raise the Malaysian pharmacist’s professional position. Pioneer-governing bodies like the Malaysian Pharmacist Society confirm that 2,889 community pharmacies are active in urban zones with their focus on the integrated healthcare system (19). Meanwhile, few suburbs and rural areas are still devoid of the same. Increasing the number of community pharmacies by providing a more convenient access would enable more screening, possibly anticipating depression earlier during occurrence. Additionally, Malay version of the PHQ-9, PHQ-2 and WHOOLEY questionnaires would guide the community pharmacists to detect more undiagnosed candidates (20, 22).

### The Community Pharmacists Symbiosis

The key to change in mental health is an effective symbiosis between the members of the multidisciplinary team. Community pharmacists ideally are the ones who form the first point of contact with a depressed patient, within the healthcare system, thus playing a triage-like role or a channel between other healthcare professionals, particularly the practicing doctors. So, it is important for the community pharmacists to spare some time and interact with such patients and furnish them with the essential information so that they can easily cope with their illness. The usage of words forms a very powerful source; an efficient communication is the one and only holy intervention, which is created for the betterment of their complex lives. To summarise, the role of community pharmacists is beyond the four walls of pharmacy and serves as a triage between the medical experts and primary care. With the synergy of committed medical workers, promising governments and conscious public, the target toward healthier Malaysia is not far away. Let’s remind that behind COVID-19 mask lays buried emotions, and being proactive, we could mend the minds.

### Future Community Pharmacists 2040

The expanded role of non-clinical community professionals may be an additional step contributing toward improving the healthcare of the depressed patients. Nevertheless, these dedicated professionals could also extend their service in screening of COVID-19 with designations redefined as ‘community-based pharmacist practitioner’; meanwhile, this second thought is still in infant stage (23). Basically, their role is focused on advancing team-based patient-care services in communities to improve the general health. In nutshell, due to their accessibility, no prior appointments, and the high trust level the general public offer them, community pharmacists are in a matchless position to aid in the opportunistic risk screening of depression.

## Figures and Tables

**Table 1:** PHQ 2+1 Questionnaire: New recommendations of USPSTF

	Over the last two weeks, how often have you been bothered by any of the following problems?	Not at all	Several days	More than half the days	Nearly every day
1.	Little interest or pleasure in doing things	0	1	2	3
2.	Feeling down, depressed or hopeless	0	1	2	3
3.	Thoughts that you would be better off dead or of hurting yourself in some way	0	1	2	3
	Add the columns	____ +	____ +	____ +	____
	Total score =				

Source: (16–17)
